# Phytochemical Profile and Evaluation of the Biological Activities of Essential Oils Derived from the Greek Aromatic Plant Species *Ocimum basilicum*, *Mentha spicata*, *Pimpinella anisum* and *Fortunella margarita*

**DOI:** 10.3390/molecules21081069

**Published:** 2016-08-16

**Authors:** Eleni Fitsiou, Gregoria Mitropoulou, Katerina Spyridopoulou, Angeliki Tiptiri-Kourpeti, Manolis Vamvakias, Haido Bardouki, Mihalis Ι. Panayiotidis, Alex Galanis, Yiannis Kourkoutas, Katerina Chlichlia, Aglaia Pappa

**Affiliations:** 1 Department of Molecular Biology and Genetics, Democritus University of Thrace, University Campus, Dragana, Alexandroupolis 68100, Greece; elenfits@gmail.com (E.F.); grigoriamitropoulou@gmail.com (G.M.); aikspiridopoulou@gmail.com (K.S.); mbg_tiptiri@yahoo.gr (A.T.-K.); agalanis@mbg.duth.gr (A.G.); ikourkou@mbg.duth.gr (Y.K.); achlichl@mbg.duth.gr (K.C.); 2 VIORYL S.A., Chemical & Agricultural Industry, Research S.A., Afidnes 19014, Greece; vamvakias@vioryl.gr (M.V.); bardouki@vioryl.gr (H.B.); 3 School of Life Sciences, Heriot-Watt University, Edinburgh, Scotland EH14 4AS, UK; M.Panagiotidis@hw.ac.uk

**Keywords:** *Ocimum basilicum*, *Mentha spicata*, *Fortunella margarita*, *Pimpinella anisum*, essential oil, composition, antimicrobial, antioxidant, antiproliferative

## Abstract

Natural products, known for their medicinal properties since antiquity, are continuously being studied for their biological properties. In the present study, we analyzed the composition of the volatile preparations of essential oils of the Greek plants *Ocimum basilicum* (sweet basil), *Mentha spicata* (spearmint), *Pimpinella anisum* (anise) and *Fortunella margarita* (kumquat). GC/MS analyses revealed that the major components in the essential oil fractions, were carvone (85.4%) in spearmint, methyl chavicol (74.9%) in sweet basil, *trans*-anethole (88.1%) in anise, and limonene (93.8%) in kumquat. We further explored their biological potential by studying their antimicrobial, antioxidant and antiproliferative activities. Only the essential oils from spearmint and sweet basil demonstrated cytotoxicity against common foodborne bacteria, while all preparations were active against the fungi *Saccharomyces cerevisiae* and *Aspergillus niger*. Antioxidant evaluation by DPPH and ABTS radical scavenging activity assays revealed a variable degree of antioxidant potency. Finally, their antiproliferative potential was tested against a panel of human cancer cell lines and evaluated by using the sulforhodamine B (SRB) assay. All essential oil preparations exhibited a variable degree of antiproliferative activity, depending on the cancer model used, with the most potent one being sweet basil against an in vitro model of human colon carcinoma.

## 1. Introduction

Aromatic plants have been extensively used in the past for culinary purposes and in traditional medicine. Nowadays, there is an increased interest in the pharmacological properties of aromatic plants that can be in part attributed to their essential oils. These are volatile mixtures of secondary metabolites, with a distinct odour, that can be extracted from all plant organs e.g., flowers, buds, stems, bark, leaves fruits, etc., and are soluble only in organic solvents. Traditionally, essential oils have been used for their biological activities, including analgesic, antiseptic, sedative, spasmolytic, anesthetic and anti-inflammatory effects. Because of their stimulant or sedative properties, they are also employed in aromatherapy [1,2].

The plant kingdom has always represented an attractive source for novel therapeutics. Among phytochemicals, essential oils, although known since antiquity, have recently regained interest due to their wide variety of bioactivities. It is only during the last decades that systematic studies have been initiated to explore, in more detail, their bioactive potential and relate it with their phytochemical profile.

In particular, their antimicrobial, antioxidant and anticancer activities are of special interest as they are associated with health-promoting properties. Currently, due to the growing health concerns of the use of synthetic antimicrobials in preventing pathogenic microbes and food spoilage, civil authorities are increasing the pressure on food manufacturers to substitute harmful synthetic preservatives with alternative natural ones. In this context, the use of essential oils with antimicrobial activity represents an attractive alternative. In addition, some essential oils possess antioxidant properties which may have a positive impact on food production by preventing oxidation [3]. On the other hand, oxidative stress is linked to many pathological conditions being the result of an imbalance between Reactive Oxygen Species (ROS) generation and their metabolism by cellular antioxidants. More specifically, oxidative stress can lead to DNA damage, mutagenicity, genotoxicity, etc., and ultimately contribute to disease development, including carcinogenesis [2,3]. Thus, compounds with antioxidant properties exert beneficial effects by protecting cells against oxidative cellular damage and thus acting as “protective shields” against carcinogenesis. It is also true that essential oils have recently gained great interest in their use as anticancer agents, as they have been found to exert their anti-proliferative potential through different mechanisms of action [4]. Conventional chemotherapy, on the other hand, is compromised by drug resistance and undesirable side-effects. Consequently, there is a need for novel agents with specific toxicity against cancer cells that will enhance the efficacy of standard treatment and may also alleviate any undesired cytotoxic side effects. Quite interestingly, studies have shown synergistic effects of conventional chemotherapeutic drugs when administered together with specific essential oils or some of their major components, responsible for exerting such effects [5,6,7,8]. This further supports the notion that nutritional intervention with natural phytochemicals, such as essential oils, may be very advantageous in enhancing the therapeutic potential of any existing therapy (e.g., chemotherapy) and furthermore diminish any adverse side effects [9].

In the present study, the essential oil volatiles of four widely used aromatic culinary Greek herbs, namely *Ocimum basilicum* (sweet basil), *Mentha spicata* (spearmint), *Pimpinella anisum* (anise) and *Fortunella margarita* (kumquat) were investigated for their chemical composition and their antimicrobial, antioxidant and antiproliferative properties in vitro. The selected plants are very popular in Greece and of high economic significance. Besides their utilization as spices, they are also used as food ingredients, and in beverages, and are well-known as home remedies in the treatment of different diseases or ailments. Thus, the purpose of this study was to further explore the potential benefits of these plants as a source of naturally occuring bioactive agents.

## 2. Results and Discussion

### 2.1. GC/MS Analysis of Essential Oils

GC/MS analysis of the essential oils is presented in Table 1. In the case of spearmint (*Mentha spicata*), a total of 12 compounds representing the 96.9% of the total chromatographic area, were identified. Carvone was the predominant compound and accounted for 85.4% of the total chromatographic area. Other major compounds identified were limonene (8.4%), and β-pinene (1.4%). Basil (*Ocimum basilicum*) analysis revealed a total of 49 compounds accounting for the 98.8% of the total chromatographic area. Methyl chavicol was the major compound (74.9%) followed by linalool (18.4%) and α-bisabolene (1.1%). For anise (*Pimpinella anisum*), 47 compounds were identified representing ~99% of the total chromatographic area. Anethole accounted for 88.1%, followed by pseudo-*iso*-eugenyl-2-methyl butyrate (4.15%) and γ-himachalene (4.15%). Finally for kumquat (*Fortunella margarita*), 45 compounds were identified, representing 99.7% of the total chromatographic area. Limonene was the principal component (93.8%). Other major compounds identified were myrcene (2.7%) and δ-germacrene (1.34%).

### 2.2. Antimicrobial Activity of Essential Oils

The antimicrobial activity of the essential oils was evaluated against six common food spoilage and pathogenic bacteria [10,11], as well as against *S. cerevisiae* and *A. niger*, which have been previously used as model systems in food spoilage and safety [12,13]. Initially, the disk diffusion method was applied for initial screening of the antimicrobial properties of essential oils, according to which the radius or diameter of the inhibition zone of microbial growth around paper disks impregnated with an antimicrobial compound is determined. Subsequently, MIC and NIC values were assessed using an established optical density method, which combines the absorbance measurements with the common dilution method, and non-linear regression analysis was used to fit the data using a previously published model [14,15]. The results are presented in Table 2, Table 3, Table 4 and Table 5.

The data obtained by the disk diffusion method indicated that all bacteria tested were sensitive to the spearmint and sweet basil essential oils (Table 2). Two initial microbial inocula were tested in order to assess growth inhibition after moderate and high contamination. Of note, no antibacterial activity was recorded for kumquat and anise essential oils. Results reporting contradictory antibacterial activity of anise and kumquat essential oils were previously published [16,17]. In contrast, all essential oils also showed considerable activity against yeasts and fungi. Large inhibition zones were observed in both *S. cerevisiae* uvaferm NEM (Table 3) and *A. niger* 19111 (the inhibition zones disappeared during incubation for longer time periods than 2 or 3 days, Table 4).

Although the inhibition zone method is widely used for the evaluation of the antimicrobial activity of essential oils, there are various factors that influence the outcome of the results, such as the composition of the sample tested (type of plant, geographical location, and time of the year), inoculum size, the ability of the essential oil to diffuse uniformly through the agar, etc. [18].

In accordance with the results of the disk diffusion method, MIC and NIC determination indicated the effective growth inhibition of both spearmint and sweet basil essential oils against all bacteria tested, although MIC and NIC values were significantly (*p* < 0.05) lower compared to ciproxin which was used as positive control (Table 5). Of note, MIC is defined as the concentration above which no growth is observed relative to the negative control test, while NIC refers to the concentration above which the inhibitor begin to display a negative effect on growth. Importantly, MIC and NIC values were significant (*p* < 0.05) lower for spearmint compared to sweet basil essential oil.

Similar results reporting considerable antimicrobial activity of the essential oils under study were previously reported [19,20,21,22]. However, other microbial species were used in most studies and MIC, and NIC values have not been estimated for all cases. Importantly, the antimicrobial activity of the essential oils could be attributed to the action of their main constituents, although possible synergistic or antagonistic effects should not be excluded [10,23]. Such effects must be further studied using model systems consisting of various mixtures of pure compounds.

### 2.3. In Vitro Antioxidant Capacity of Essential Oils

The essential oils were tested for their ability to scavenge the free radicals generated by DPPH and ABTS methods. The results indicate that essential oils possess weak in vitro antioxidant capacity (Table 6). The oils caused a maximum of 48% inhibition of the DPPH radical (anise oil), in the case of kumquat this percentage was further reduced to 34.5% and for basil inhibition reached only 14.5%. For the study of spearmint oil, the maximum concentration that could be tested was approximately ten times lower than the other oils (4.8 mg/mL) because significant turbidity was observed at higher concentrations of the reaction solution that interfered with the measurement of the absorbance. At the given concentration, DPPH inhibition was 6%.

As far the ABTS method is concerned, the highest activity was shown by spearmint oil (53.2% inhibition) followed by basil oil (43.7% inhibition), while anise and kumquat oil were the least potent (18.6% and 6.7% inhibition, respectively). These results differ from the results of the DPPH assay; however, this difference in the behavior of the oils has been reported before. The antioxidant activity of the essential oil of *Cedrelopsis grevei* was found to be higher using the ABTS assay compared to the DPPH assay [24], while different essential oils from Burkina Faso exhibited diverse results between the two methods [25]. Similarly, Yu et al., showed that three wheat varieties exhibited different capacity to quench DPPH and ABTS [26]. These differences can be attributed to a variety of factors, such as stereoselectivity of the radicals, solubility of the oils in the different systems, and mechanism of action of the reaction or the antioxidant [24,25,26]. For example, limonene (the main component of kumquat) has been found to scavenge the DPPH radical more effectively than the ABTS cation, whereas methyl chavicol (the main component of sweet basil oil), reacted more efficiently with ABTS [27]. This is in agreement with our results, where kumquat oil caused higher DPPH inhibition, while basil oil was more potent against ABTS.

Sweet basil oil is the most studied among the other essential oils that we present. Interestingly, the essential oil of basil has shown great variation depending on its geographical origin, which may explain reported differences in its biological properties [28]. It has shown moderate antioxidant activity compared to other *Ocimum* species which was, however, better than the activity of olive and sesame oils. The essential oil of sweet basil with linalool and α-terpineol as major components exhibited significant antioxidant activity in vitro [25,29,30,31]. An oil isolated from Iranian plants showed potent antioxidant capacity and had methyl chavicol as major component, although in a lower percentage compared to the oil we isolated (47% vs. 75%) [32], while Dawidowicz and Olszowy showed that the antioxidant activity of the oil was not attributable to its major component, as methyl chavicol did not neutralize DPPH [27].

Spearmint has been shown to possess significant antioxidant activity in vitro, which may be due to differential content in carvone compared to our oil [33]. Interestingly, when spearmint oil was used as a supplement in the diet of rainbow trout juveniles for two months, although antioxidant enzymes levels were increased, the oil affected growth parameters and survival, indicating it should be used as an additive with caution [34]. Kumquat oil has shown significant antioxidant activity, maybe due to its lower content in limonene [35]. Finally, anise oil demonstrated significant antioxidant activity using different methods in vitro [36]. As stated in the study, there are many factors that can affect the activity of oils such as harvesting time, extraction process and environmental factors (location, altitude, soil, light, temperature, wind, climate, stress exposure, etc.).

### 2.4. Antiproliferative Activity of Essential Oils

For the determination of the antiproliferative activity of the oils against a panel of human cancer cell lines after a 72 h incubation, the SRB assay was used for HepG2, Caco2 and MCF-7 cells, which is based on the ability of sulforhodamine B dye to bind electrostatically to basic amino acid residues of acid-fixed cells, while the XTT method was used for THP-1 cells The results are presented in Table 7.

**Figure 1:**
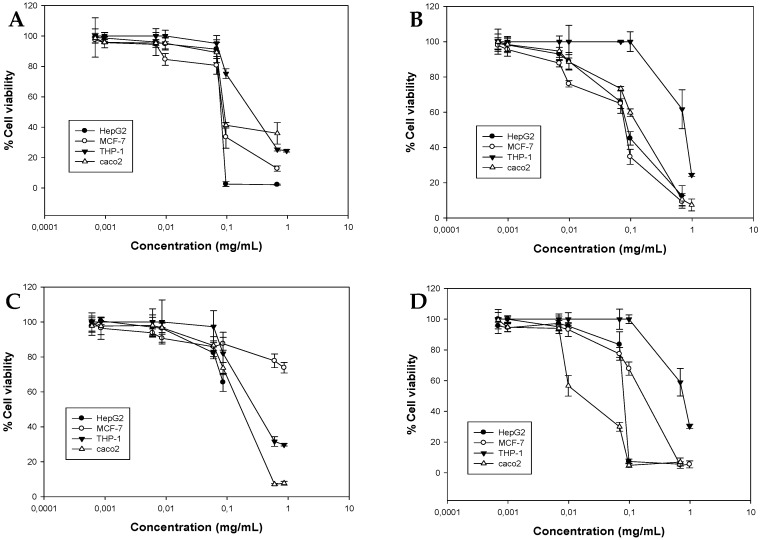
Antiproliferative activity of (**A**) anise (**B**) spearmint (**C**) kumquat (**D**) sweet basil oils against a panel of four human cancer cell lines. Cancer cells were incubated with increasing concentrations of the oils for 72 h. Estimation of cell viability was determined by the SRB assay. Representative figures of at least three experiments.

The viability curves for the essential oils are shown in Figure 1. Each oil differed in its activity against the cancer cell lines used in this study and presented a unique pattern of cytotoxicity; however, all oils exhibited moderate to weak cytotoxicity compared to potent chemotherapeutic agent etoposide which was used as positive control (Table 7). In general, spearmint oil was the least potent against the THP-1 cells line (EC_50_ = 0.71 ± 0.004 mg/mL), while it exhibited similar activity against HepG2 and MCF-7 cells line (EC_50_ = 0.22 ± 0.038 mg/mL and EC_50_ = 0.284 ± 0.02 mg/mL, respectively). Caco2 cells were the most sensitive to spearmint oil (EC_50_ = 0.162 ± 0.0035 mg/mL) (Figure 1B). Like spearmint oil, anise had similar activity against HepG2 and MCF-7 cells (EC_50_ = 0.39 ± 0.0282 mg/mL and EC_50_ = 0.3 ± 0.01 mg/mL, respectively) and was most cytotoxic against THP-1 cells (EC_50_ = 0.11 ± 0.00067 mg/mL) (Figure 1A). Kumquat oil did not affect the viability of HepG2 and MCF-7 cells significantly, causing less than 50% reduction in cell number, while Caco2 and THP-1 showed similar sensitivity to the action of the oil (Figure 1C). Sweet basil was most cytotoxic against Caco2 cells, followed by HepG2 and MCF-7 cells where the EC_50_ values were similar (Figure 1D).

It is worth mentioning that sweet basil oil exhibited the most potent action against HepG2, MCF-7 and Caco2 cells compared to the other oils. THP-1 cells were the most resistant to the oil, where there was a seven- to nine- fold increase in the EC_50_ value in comparison to the other cell lines. Anise oil from Iranian plants was able to reduce the viability of HepG2 cells by more than 50% at a concentration <100 μg/mL after 24 h incubation, but had no significant protective effect against induced hepatotoxicity in vitro and in vivo in non-toxic concentrations [37], while kumquat oil demonstrated significant antiproliferative activity against LNCaP cells.

Sweet basil as well as spearmint belong to the *Lamiaceae* family and many essential oils from plants of this family have also received great attention and studied for their antioxidant and antiproliferative or possible synergistic effects with conventional chemotherapeutic drugs [38,39,40]. To our knowledge this is the first time that the antiproliferative activity of sweet basil oil against the human cancer cell lines HepG2, Caco2, MCF-7, and THP-1 is determined. However, there are data showing its potent cytotoxicity following different incubation times and against cancer cell lines of different types including human prostate, glioblastoma, laryngeal, cervical and mouth and also murine leukemia, but was also cytotoxic to normal mouse embryonic fibroblasts [11,25,28,41].

In another study, a Chinese commercial spearmint oil preparation did not affect the viability of MCF-7 and A549 cells, although it was cytotoxic against the androgen-independent cancer cell line PC-3 in the range of concentrations tested [42]. Spearmint oil was also found to be a potent antiproliferative agent against KB, P388, LNCaP and MCF-7 cells after 24 h of incubation [41,43].

## 3. Materials and Methods

### 3.1. Essential Oil Extraction and GC/MS Analysis

Essential oils were obtained at the VIORYL facilities by hydrodistillation. Chopped leaves and stems of the plant material were used for the species *Ocimum basilicum* (collected during the months of May and June), and *Mentha spicata* (collected during spring and autumn) without further drying. Seeds of *Pimpinella anisum* (collected during mid-summer) and the chopped peel of *Fortunella margarita* fruits (collected on the island of Corfu between January and March) were directly treated and processed for hydrodistillation. Following decantation, essential oils were dried over anhydrous sodium sulfate. In all cases, hydrodistillation took place immediately after the harvesting period (respecting seasonality restrictions) so that the plants/seeds/fruit peels would provide the most of their essential oils. A Dean Stark apparatus was used for hydrodistillation [44] where the studied material was placed along with 6 L of distilled water. After hydrodistillation (8 h, 90–120 °C), the essential oil was isolated. Subsequently samples were dried with Na_2_SO_4_ and collected to sealed vials for further use. GC/MS analysis was carried out with a GC-MS (GC: 6890A and MSD: 5973, Agilent Technologies, Santa Clara, CA, USA) using a Factor Four VF 1 ms column (25 m, 0.2 mm i.d., 0.33 μm film thickness, Agilent Technologies). Essential oil (0.1 μL) was directly injected and a 1:100 split ratio was applied. The oven temperature was set at 50 °C for 1 min, followed by a temperature gradient of 2.5 °C/min to 160 °C for 20 min and then 50 °C/min to 250 °C for 15 min. Helium was used as carrier gas (flow rate 1 mL/min). Injector and transfer line temperatures were set to 200 °C and 250 °C, respectively. The mass spectrometer operated in the electron impact mode with the electron energy set to 70 eV. Identification of the compounds was carried out according to the standard method of Kováts Indices.

### 3.2. Microbial Strains

*Salmonella enterica* subsp. *enterica* ser. Enteritidis FMCC Β56 PT4 (kindly provided by G.J.E. Nychas, Agricultural University of Athens, Athens, Greece), *Salmonella enterica* subsp. *enterica* ser. Typhimurium DSMZ 554, *Listeria monocytogenes* NCTC 10527 serotype 4b, *Escherichia coli* ATCC 25922, *Staphylocccus epidermidis* FMCC B-202 C5M6 (kindly provided by Nisiotou A., Wine Institute of Athens, ELGO “DEMETER”, Lykovrysi, Greeceand *Staphylococcus aureus* ATCC 25923 were grown in Brain Heart Infusion (BHI) broth (LABM, Heywood, UK) at 37 °C for 24 h. *Saccharomyces cerevisiae* uvaferm NEM (Lallemand, Montreal, QC, Canada) was grown in YPD broth (yeast extract 10 g/L, glucose 20 g/L and peptone 20 g/L) at 28 °C for 3 days. *Aspergillus niger* 19111 (kindly provided by G.J.E Nychas) was grown on malt extract agar (LABM) for 7 days at 37 °C. 

### 3.3. Antimicrobial Assays

The antimicrobial activity of the tested essential oil was monitored using the two following methods [45].

#### 3.3.1. Disk Diffusion Assay

For the antibacterial screening, the disk diffusion assay was performed. The bacterial suspensions were 10-fold diluted in ¼ Ringer’s solution (LABM). A 0.1 mL portion from the appropriate dilution was spread on Brain Heart Infusion (BHI) agar (LABM), in order to provide initial inoculums of 10^5^ or 10^7^ cfu/mL. Subsequently, sterile paper disks (Whatman No. 2) of 5 mm diameter were placed onto the inoculated agar surface containing 5 μL (4700 μg spearmint, sweet 4600 μg basil, 4200 μg kumquat, 4800 μg anise) of the essential oils. Petri dishes were incubated at 37 °C for 24 h. After incubation, the diameter of the inhibition zones were measured in mm. The same procedure was also followed for the screening of the activity against yeasts, using *S. cerevisiae* suspensions 10-fold diluted in ¼ Ringer’s solution (LABM) and spread on YPD agar, which were then incubated at 28 °C for 3 days and then the inhibition zones were measured in mm. For the antifungal activity, 100 fungal spores/plate from *A. niger* were spread on Malt Extract agar (LABM) and the above procedure was followed. The diameter of the inhibition zones were measured daily in petri dishes were incubated at 37 °C for 10 days. Ciproxin (5 μg) (Oxoid Ltd., Basingstoke, UK) was used as positive control for bacteria and amphotericin B (10 μg) (Mast Group Ltd., Merseyside, UK) for yeast and fungi. Sterile water was used as negative control. All experiments were carried out at least in triplicate, and the mean values are presented.

#### 3.3.2. Determination of Minimum Inhibitory Concentration (MIC) and Non-Inhibitory Concentration (NIC)

Determination of MIC and NIC values was carried out as recently described [45]. In brief, bacterial growth in BHI broth (LABM) was monitored through changes in optical density of bacterial suspensions in the presence of multiple concentrations of essential oils. Stock solutions (ranging 43–9300 mg/L) of the essential oils were prepared by mixing them directly with BHI broth. Aliquots (0.180 mL) of growth medium mixed with the essential oils were transferred to the wells of a 96-well microplate. The bacterial suspensions were diluted tenfold in ¼ Ringer’s solution and a 0.070 mL portion from the appropriate dilution was added to the wells containing the growth medium (final volume 0.250 mL), in order to result in a population of approximately 10^3^ cfu/mL. Microplates were incubated in Microplate Reader (VERSAmax, Molecular Devices, Sunnyvale, CA, USA, Softmaxpro v. 5.0 software) at 37 °C for 24 h. Optical density measurements were carried out every 10 min at 610 nm. Ciproxin (positive control) stock solutions (0.5–4 mg/L) were prepared by mixing the antibiotic directly with BHI broth. BHI broths with no inoculum and inoculated BHI broths with no essential oils were used as negative controls. The calculation of MIC and NIC values was based on the Lambert-Pearson model (LPM) [14,15]. In brief, the effect on the growth, measured by the optical density method, is manifested by a reduction in the area under the OD/time or curve relative to control well at any specified time. By calculating the area using the trapezoidal rule, the relative amount of growth were obtained using the ratio of the test area to that of the control, termed the fractional area, fa. Data were fitted to the LPM using non-linear least squares regression analysis assuming equal variance.

### 3.4. Cell Lines and Cell Cultures

The human hepatocellular carcinoma HepG2, the human breast adenocarcinoma MCF-7, the human colon adenocarcinoma Caco2 and the human leukemic monocytic THP-1 cell lines were obtained from the American Type Culture Collection (Rockville, MD, USA). HepG2 and MCF-7 cells were grown and maintained in Dulbecco’s modified Eagle’s medium (DMEM, Gibco, Waltham, MD, USA) while Caco2 and THP-1 cells were cultured and maintained in RPMI-1640 medium (Gibco), both supplemented with 10% fetal bovine serum (FBS), and penicillin (100 U/mL) (Biosera, Boussens, France) and were incubated at 37 °C in a humidified atmosphere of 95% O_2_ and 5% CO_2_. Stock cultures were passaged at 2- to 3-day intervals. Cells were seeded at a density of 3.0–5.0 × 10^3^ cells/well in 96-well plates for the sulforhodamine B (SRB) assay. THP-1 cells were seeded at a density of 2.0 × 10^3^ cells/well in round bottom 96-well plates for the XTT assay.

### 3.5. Antioxidant Activity

#### 3.5.1. DPPH Assay

The radical scavenging activity of the essential oils was estimated using the free radical 2,2-diphenyl-1-picrylhydrazyl (DPPH), as described previously with few modifications [46]. Different concentrations of the essential oils (basil oil (0.0049–49 mg/mL), anise oil (0.00485–48.5 mg/mL), kumquat oil (0.0043–43 mg/mL) and spearmint oil (0.0048–4.8 mg/mL)) were prepared using dimethyl sulfoxide (DMSO, Biotium, Fremont, CA, USA) as the solvent. Ten microliters of each concentration were placed in a 96-well plate, and 190 μL of 300 μM methanolic solution of DPPH (Calbiochem, Darmstadt, Germany) was added. Ten microliters of DMSO with 190 μL DPPH was used as the control. Ascorbic acid was used as a positive control (Sigma-Aldrich, St. Louis, MO, USA). The plate was left in darkness for 30 min, and then the absorbance was measured at 517 nm using an Elisa plate reader (EnSpire Multimode Plate Reader, Perkin Elmer, Waltham, MA, USA). The % inhibition of the DPPH radical for each concentration was determined by making use of the following formula: % DPPH radical scavenging activity = [(OD_control_ − OD_sample_)/OD_control_)] × 100.

#### 3.5.2. ABTS Assay

The ABTS [2,2′-azino-bis(3-ethylbenzothiazoline-6-sulphonic acid)] decoloration assay was performed as described previously with few modifications [47]. Seven mmoles of ABTS (Sigma-Aldrich) dissolved in water were mixed with 2.45 mM potassium persulfate (final concentration, Sigma-Aldrich), and were let to stand in the dark for 16 h in order to allow the formation of the ABTS radical cation (ABTS^•+^). The cation was further diluted in ethanol (Scharlau, Barcelona, Spain) in order to obtain absorbance of 0.8 at 734 nm. Different concentrations of the essential oils were prepared in DMSO. Ten microliters of each concentration were placed in a 96-well plate, and 190 μL of ABTS^•+^ was added. Ten microliters of DMSO with 190 μL ABTS^•+^ was used as the control. The plate was left in darkness for 15 min, and then the absorbance was measured at 734 nm using an Elisa plate reader (EnSpire Multimode Plate Reader, Perkin Elmer) against a standard curve with ascorbic acid. The % inhibition of the ABTS^•+^ cation for each concentration was determined by making use of the following formula: % ABTS^•+^ radical scavenging activity = [(OD_control_ − OD_sample_)/OD_control_)] × 100. Furthermore, the results are expressed as micromoles ascorbic acid equivalent per gram of essential oil (mmolEA/g).

### 3.6. Cell Viability Assays

#### 3.6.1. Sulforhodamine B Assay

The viability of the cancer cell HepG2, Caco2, and MCF-7 after treatment with the essential oils was determined using the SRB assay. SRB is a dye that binds to basic amino acids of cellular proteins and, then, the number of viable cells is estimated with colorimetric evaluation [48]. Cells were plated in 96-well plates and treated with different concentrations of the essential oils [basil oil (0.00068–0.98 mg/mL), anise oil (0.00068–0.97 mg/mL), kumquat oil (0.0006–0.86 mg/mL) and spearmint oil (0.00067–0.96 mg/mL)] (dissolved in DMSO, 1:1 *v*/*v*) for 72 h. The anticancer drug etoposide (Sigma-Aldrich) was used a positive control. Then, the cells were fixed with the addition of 25 μL of 50% (*w*/*v*) cold trichloroacetic acid (TCA) (MP Biomedicals, Santa Ana, CA, USA) to the growth medium and incubation of the plates at 4 °C for 1 h. The cells were washed five times with tap water and then stained with 50 μL of 0.4% (*w*/*v*) SRB (Sigma-Aldrich) in 1% (*v*/*v*) acetic acid (Scharlau) for 30 min at room temperature. Then, the cells were rinsed five times with 1% (*v*/*v*) acetic acid to remove the unbound dye. The fixed, stained plates were allowed to air-dry followed by solubilization of the bound dye by adding 100 μL of 10 mM Trizma base (Sigma-Aldrich) for at least 5 min. Absorbance was measured at 570 nm using an Elisa plate reader (EnSpire Multimode Plate Reader, Perkin Elmer), and the percent cellular survival was calculated using the formula: [(sample OD_570_ − media blank OD_570_)/(mean control OD_570_ − media blank OD_570_)] × 100.

#### 3.6.2. XTT Cell Viability Assay

The viability of THP-1 cells was determined by the XTT (2,3-bis(2-methoxy-4-nitro-5-sulfophenyl)-*S*-(phenylamino)carbonyl-2-tetrazolium hydroxide) assay [49]. In all experiments, the XTT Cell Viability kit (Cell Signaling, Danvers, MA, USA) was used according to the manufacturer’s protocol. Briefly, cells were seeded in a 96-well-plate. After overnight incubation, cells were treated with increasing concentrations of essential oils (dissolved in DMSO, 1:1 *v*/*v*) for 72 h. Control cells were treated with DMSO-containing medium at concentration <0.1% *v*/*v*. The anticancer drug etoposide (Sigma-Aldrich) was used a positive control. At the end of the incubation, the XTT solution was added, and plates were placed in the incubator for 4 h and then absorbance was measured at 450 nm with a microplate reader (EnSpire Multimode Plate Reader, Perkin Elmer).

### 3.7. Data Analysis

All experiments were performed at least in triplicate. For MIC and NIC determination, each experiment was performed at least 4 times and standard deviation was calculated by Fig. P software (Fig.P Software Incorporated, Hamilton, ON, Canada). Significance was established at *p* < 0.05 and the results were analyzed for statistical significance with analysis of variance (ANOVA). Duncan’s multiple range test was used to determine significant differences among results (coefficients, ANOVA tables and significance (*p* < 0.05) were computed using Statistica v.5.0). The EC_50_ values (Effective Concentration; the concentration of test samples required to cause decrease of cancer cell viability by 50%) were calculated from the respective dose-response curves by regression analysis using a four-parameter logistic curve through the Sigma Plot Software v.10 (Systat Software Inc., San Jose, CA, USA).

## 4. Conclusions

The present work reports a comparative study of the chemical composition and the biological potential of essential oil volatiles from four widely used aromatic plants: *Mentha spicata*, *Ocimum basilicum*, *Pimpinella anisum* and *Fortunella margarita*, all grown in Greece. Chemical analysis by GC/MS showed that carvone, methyl chavicol, *trans*-anethole and limonene were the major components of *Mentha spicata*, *Ocimum basilicum*, *Pimpinella anisum* and *Fortunella margarita*, respectively. All essential oil preparations showed activity against the fungi *Saccharomyces cerevisiae* and *Aspergillus niger*, but only *Mentha spicata* and *Ocimum basilicum* were cytotoxic against common foodborne bacteria. Antioxidant evaluation by DPPH and ABTS radical scavenging activity assays revealed a variable degree of antioxidant potency. All essential oil preparations exhibited antiproliferative activity that also varied depending on the cancer model used, with the most potent one being *Ocimum basilicum* against an in vitro human colon carcinoma model. Further studies are required to correlate specific biological properties with active chemical components and/or possible compound synergy effects. In conclusion, it is of great interest to screen commonly used plants from the local flora for potent biological activities. Besides being safe (widely used for generations) and easily available, they could represent a new alternative source of bioactive substances for various applications in the food, pharmaceutical, neutraceutical and other industries.

## Figures and Tables

**Table 1:** Compounds identified in the volatiles of essential oils by GC/MS and their relative percent (%) area.

Compounds	KRI *	*Mentha spicata* (% Area)	*Ocimum basilicum* (% Area)	*Pimpinella anisum* (% Area)	*Fortunella margarita* (% Area)
*cis*-3-Hexenol	811		0.014		Trace
Thujene	915				Trace
α-Pinene	922	0.670	0.069	0.081	0.743
Camphene	927				Trace
Sabinene	953			0.060	0.133
Methyl heptenone	954		0.134		
Oct-1-en-3-ol	955		0.001		
β-Pinene	958	1.450	0.038	0.054	0.019
Myrcene	973		0.039	0.010	2.680
α-Phellandrene	981			0.089	0.073
δ-3-Carene	990				0.020
α-Terpinene	997				Trace
*p*-Cymene	1004		0.001	0.088	Trace
β-Phellandrene	1004			0.016	
2-Ethylhexenol	1006		0.134		
1,8-Cineole	1010		0.020		
Limonene	1011	8.410	0.020	0.035	93.784
*cis*-Ocimene	1016			0.016	0.001
*trans*-Ocimene	1018		0.001	Trace	0.019
γ-Terpinene	1030		0.061	0.034	0.023
Epoxylinalool I	1049		0.167	Trace	
Alc C8	1050		0.001		
Thujone	1057		0.001		
Dehydro-*p*-cymene	1062			0.013	Trace
Epoxylinalool II	1064		0.149		
Terpinolene	1070			0.046	0.014
Linalol	1086		18.400	0.278	0.118
Octen-1-en-3-yl acetate	1087		0.001		
*trans*-*p*-Menthene-2.3-dien-1-ol	1105				0.018
Camphor	1108			0.022	
*cis*-*p*-Menthene-2.8-diene-1-ol	1115				0.017
*p*-vinylanisole	1118			0.017	
Menthone	1124	0.130	0.033		
*iso*-Menthone	1133	0.040	0.017		
Borneol	1138			Trace	
Menthol	1150	0.190	0.240		
*p*-Cymenol	1151			0.016	
Terp-1-ene-4-ol	1152				0.020
Dihydrocarvone	1160	0.200			
Dihydrocarveol	1160	0.130			
α-Terpineol	1168		0.003	0.012	0.026
3-Hexenyl butyrate	1168		0.003		
Epoxyphellandrene	1171			Trace	
8-Cumenol	1172				Trace
*trans*-Carveol	1177				0.014
Methyl chavicol	1177		74.920	1.525	
Decanal	1178				0.015
Octyl acetate	1191		0.028		0.055
*cis*-Carveol	1197				0.011
Nerol	1203		0.040		
Neral (*cis*-citral)	1205		0.200		
Anisaldehyde	1207		0.110	0.545	
Carvone	1217	85.410			0.023
Piperitone	1218		0.001		
*cis*-Anethole	1218			0.435	
Geraniol	1231				Trace
Perilla aldehyde	1233				0.019
Geranial (*trans*-citral)	1237		0.519		
*trans*-Anethole	1265		0.028	88.130	
Isobornyl acetate	1277		0.001		
Dihydrocarvenyl acetate	1304	0.130			
δ-Elemene	1327			0.149	0.022
Eugenol	1331		0.059		
Anisyl methyl ketone	1339			0.025	
α-Longipinene	1339			0.061	
Neryl acetate	1340				0.014
α-Cubebene	1344				Trace
Cyclosativene	1357			0.041	
Geranyl acetate	1358				0.111
Ylangene	1360			0.050	
Methyl eugenol	1365		0.049		
α-Copaene	1366		0.029		0.016
β-Bourbonene	1371	0.040	0.019	0.033	
β-Elemene	1378			0.098	0.023
*p*-Menth-1-en-9-yl acetate	1399				0.007
Caryophyllene	1403	0.070	0.273		0.010
Methoxypropiophenone	1402			0.048	
Bergamotene	1424		0.509		
a Farnesene	1427		0.054	0.054	
α-Himachalene	1431			0.381	
Humulene	1436		0.154		0.008
*cis*-β-Farnesene	1438		0.219		
Dehydro-*neo*-isolongifolene	1443			0.079	
Methyl-isoeugenol	1446			0.088	
ar-Curcumene	1460		0.025	0.091	
γ-Himachalene	1460			4.155	
δ-Germacrene	1462		0.025		1.343
*trans*-β-Farnesene	1468		0.025		
Zingiberene	1478			0.570	
Bicyclogermacrene	1479				0.246
β-Chimachalene	1481			0.243	
α-Mourolene	1483				Trace
Myristicin	1487			0.045	
β-Bisabolene	1492		0.097	0.473	
Calamenene	1496			0.019	
Valencene	1501				0.009
δ-Cadinene	1504			0.091	0.053
*p*-Methoxycinnamic ald	1507		0.572		
α-Calacorene	1516			Trace	
α-Bisabolene	1525		1.068		
β-Germacrene	1533				0.039
Caryophyllene oxide	1551		0.135		
1,5,5,8-Tetramethyl-12-oxabicyclo[9.1.0]dodeca-3.7-diene	1575		0.053		
Pseudo-isoeugenyl-2-methyl butyrate	1833			4.155	

* Kováts Retention Indices.

**Table 2:** Antibacterial activity of the essential oils against common food spoilage and pathogenic bacteria monitored by the disk diffusion assay. Ciproxin was used as positive control.

Essential Oil	5 log cfu/mL Initial Inoculum					
	*S. enteritidis*	*S. typhimurium*	*E. coli*	*S. epidermidis*	*S. aureus*	*L. monocytogenes*
Spearmint	14 ± 0.5	13 ± 0.5	13 ± 0.3	15 ± 0.3	13 ± 0.3	11 ± 0.5
Sweet basil	12 ± 0.5	13 ± 0.7	13 ± 0.5	17 ± 0.3	15 ± 0.5	13 ± 0.3
Kumquat	0	0	0	0	0	0
Anise	0	0	0	0	0	0
Ciproxin	31 ± 0.3	37 ± 0.3	34 ± 0.5	35 ± 0.3	33 ± 0.3	33 ± 0.5
**Essential Oil**	**7 log cfu/mL Initial Inoculum**					
	***S. enteritidis***	***S. typhimurium***	***E. coli***	***S. epidermidis***	***S. aureus***	***L. monocytogenes***
Spearmint	10 ± 0.5	10 ± 0.5	10 ± 0.5	10 ± 0.5	10 ± 0.5	10 ± 0.5
Sweet basil	10 ± 0.5	10 ± 0.5	10 ± 0.5	10 ± 0.5	10 ± 0.5	10 ± 0.5
Kumquat	0	0	0	0	0	0
Anise	0	0	0	0	0	0
Ciproxin	25 ± 0.5	25 ± 0.3	30 ± 0.5	25 ± 0.5	26 ± 0.3	23 ± 0.3

The diameter of the inhibition zones were measured in mm.

**Table 3:** Antimicrobial activity of the essential oils against *Saccharomyces cerevisiae* monitored by the disk diffusion assay. Amphotericin B was used as positive control.

Essential Oil	Inoculum (log cfu/mL)	
	5	7
Spearmint	35 ± 0.5	27 ± 0.5
Sweet basil	20 ± 0.7	16 ± 0.7
Kumquat	29 ± 0.7	24 ± 0.5
Anise	16 ± 0.7	13 ± 0.5
Amphotericin B	24 ± 0.3	20 ± 0.3

The diameter of the inhibition zones were measured in mm.

**Table 4:** Antifungal activity of the essential oils and amphotericin B as positive control against *Aspergillus niger* monitored by the disk diffusion assay.

Essential Oil	1 Day	2 Days	3 Days
Spearmint	40 ± 0.5	25 ± 0.5	0
Sweet basil	15 ± 0.5	10 ± 0.7	0
Kumquat	18 ± 0.3	0	0
Anise	40 ± 0.7	20 ± 0.5	0
Amphotericin B	22 ± 0.5	20 ± 0.5	19 ± 0.3

The diameter of the inhibition zones were measured daily in mm.

**Table 5:** MICs and NICs (mg/L) of the essential oils against common food spoilage and pathogenic bacteria.

Microbial Species	Spearmint		Sweet Basil		Ciproxin	
	MIC	NIC	MIC	NIC	MIC	NIC
*S. enteritidis*	1960 ± 9	600 ± 9	4270 ± 29	2000 ± 20	0.976 ± 0.001	0.957 ± 0.001
*S. typhimurium*	3670 ± 22	1280 ± 29	3880 ± 33	2660 ± 12	0.979 ± 0.001	0.964 ± 0.001
*E. coli*	1980 ± 33	580 ± 11	2410 ± 10	1500 ± 19	0.984 ± 0.001	0.956 ± 0.002
*S. epidermidis*	2590 ± 14	610 ± 20	4190 ± 23	1570 ± 10	0.979 ± 0.002	0.957 ± 0.002
*S. aureus*	2530 ± 20	650 ± 20	5720 ± 20	1020 ± 11	0.982 ± 0.002	0.963 ± 0.003
*L. monocytogenes*	2480 ± 15	710 ± 12	5369 ± 29	1650 ± 18	0.978 ± 0.001	0.968 ± 0.002

**Table 6:** Antioxidant activity of the essential oils in the maximum concentration tested using the DPPH and ABTS assays.

Essential Oil (Highest Concentration Used)	% DPPH Inhibition	% ABTS Inhibition	ABTS (μmolesEA/g) *
Kumquat (43 mg/mL)	34.5 ± 0.07	6.7 ± 0.1	326.2 ± 0.05
Spearmint (4.8 mg/mL)	6 ± 1.45	53.2 ± 0.02	9833.3 ± 10.5
Basil (49 mg/mL)	14.5 ± 0.01	43.7 ± 0.03	834.3 ± 3.4
Anise (48.5 mg/mL)	48 ± 0.07	18.6 ± 0.03	383.5 ± 6
Ascorbic acid (0.11 mg/mL)	76.5 ± 0.002	96.5 ± 0.001	-

Representative results from at least three independent experiments; * micromoles ascorbic acid equivalent per gram of essential oil.

**Table 7:** EC_50_ values of the essential oils against the different human cancer cell lines tested. Etoposide was used as a positive control.

	EC_50_ (mg/mL)			
	HepG2	Caco2	MCF-7	THP-1
Sweet basil	0.18 ± 0.028	0.071 ± 0.0032	0.17 ± 0.022	0.67 ± 0.00214
Kumquat	n.d.	0.1 ± 0.027	n.d.	0.1 ± 0.0023
Spearmint	0.22 ± 0.038	0.162 ± 0.0035	0.284 ± 0.02	0.71 ± 0.004
Anise	0.39 ± 0.0282	0.25 ± 0.04	0.3 ± 0.01	0.11 ± 0.00067
Etoposide	0.00065 ± 0.000063	0.0073 ± 0.00063	0.00167 ± 0.00041	0.00045 ± 0.000013

Data are presented as Mean ± SD of at least three independent experiments. n.d. = not determined.
